# Hypoglycemic and Toxic Effect of *Morus mesozygia* Leaf Extract on the Liver and Kidneys of Alloxan-Induced Hyperglycemic Wistar Rats

**DOI:** 10.1155/2019/6712178

**Published:** 2019-09-19

**Authors:** Michael Tirwomwe, Isaac Echoru, Richard Maseruka, Kyobe Ronald Kimanje, Wilson Byarugaba

**Affiliations:** ^1^Department of Biomedical Sciences, School of Medicine, Kabale University, Kabale, Uganda; ^2^Department of Biochemistry, Faculty of Biomedical Sciences, Kampala International University, Kampala, Uganda; ^3^Department of Anatomy, Faculty of Biomedical Sciences, Kampala International University, Kampala, Uganda; ^4^Department of Biology, Faculty of Science, Kabale University, Kabale, Uganda

## Abstract

**Purpose:**

We investigated the hypoglycemic and toxic effect of *Morus mesozygia* leaf extract on the liver and kidneys of alloxan-induced hyperglycemic wistar rats.

**Method:**

Phytochemical analysis was done. Diabetes was induced by the use of alloxan monohydrate in six groups of rats, i.e., 200 mg/kg, 400 mg/kg, 800 mg/kg, glibenclamide, normal saline, and normal control group. Blood glucose was measured at the time of inoculation, then at 1, 2, 3, and 4 hours after. After 14 days, rats were killed under anesthesia; blood collected for measurement of total protein, albumin, TAGs, cholesterol, AST, ALT, urea, and creatinine; and whole tissue of liver and kidneys used for histological studies.

**Results:**

The extract possessed antidiabetic effects between 400 mg/kg and 800 mg/kg doses, which we attributed to the presence of flavonoids, tannins, terpenoids, and amino acids. There was a drop in total protein and albumin with no statistical significance (*P* ≥ 0.05). The changes in levels of ALT, TAGs, cholesterol, AST, creatinine, and urea were not statistically different from the standard diabetic drug. The extract was protective against histological damage as there were no significant lesions suggestive of toxicities in the liver and kidneys at doses below 800 mg/kg.

**Conclusion:**

We established credible evidence that *Morus mesozygia* leaf extract has hypoglycemic effects between 400 mg/kg and 800 mg/kg and that it is safe on the liver and kidneys of wistar rats at doses less than 800 mg/kg.

## 1. Introduction

Diabetes mellitus (DM) is one of the leading causes of mortality and morbidity in developed and developing countries. DM is characterized by increased levels of glucose that eventually progress to frequent urination, increased thirst, and increased hunger [1]. According to global estimates of diabetes, in 2013, 382 million people had diabetes; this number is however expected to rise to 592 million by 2035. Majority of the people with diabetes live in low- and middle-income countries and these will experience the greatest increase in cases of diabetes over the next 22 years [2]. In Africa alone, about 12.1 million people lived with diabetes mellitus by 2010 [3, 4], and according to research, there will be an expected increment in the prevalence of DM in Asia and Africa by 2030. This is because of increased urbanization and lifestyle changes that have changed the indigenous diet to a modern diet [5].

A recent study showed that the prevalence of diabetes was 16.1% (*n* = 200) from rural communities in Uganda [6]. A study of 454 adults in Western Kenya showed 180 (40%) patients had symptoms suggestive of DM [7].

Today, different types of DM are managed by the use of insulin therapy and other oral hypoglycemic agents; however, high cost, poor availability, and undesirable side effects of these synthetic drugs have led many rural communities to rely on drugs of plants origin [8–10]. In western Uganda, many patients with symptoms related to diabetes, such as polydipsia, fatigue, and decreased sensitivity in lower limbs, seek traditional medicine for reasons such as high costs and failure of effectiveness from western medicine [9]. They use several herbal extracts because they are claimed to be less toxic and free from side effects in comparison to synthetic ones [11].

Uganda is endowed with a rich plant ethnomedicinal tradition. Several herbal preparations possessing hypoglycemic effects have been used in the management of diabetes mellitus [12]. Research has shown that medicinal plants with antidiabetic effects exhibit biochemical mechanisms such as restoration of pancreatic *β*-cell function, improvement in insulin sensitivity by receptors, stimulating the rate of insulin secretion, inhibition of liver gluconeogenesis, enhancement of glucose absorption, and inhibition of G-6-Pase, *α*-amylase, and *α*-glucosidase activities [13].

One of the most popular plants used in the management of DM in western Uganda is the mulberry tree (*Morus mesozygia*), commonly known as *enkyerere*. Mulberry leaves are used to treat hyperglycemia, inflammation, cough, hypertension, cancer, and fever. The efficacy of the plant in curing debilitating diseases has been attributed to the presence of flavonoids, anthocyanins, and alkaloids in the leaves, bark, root, and fruits [14, 15].

Worldwide, mulberry is grown for its fruit and is believed to have medicinal properties. The fruit and leaves are sold in various forms as nutritional supplements. The unripe fruit and green parts of the plant have a white sap that is intoxicating and slightly hallucinogenic. Anthocyanins found in mulberries are pigments that hold potential use as dietary modulators of mechanisms for various diseases. The species commonly distributed in Africa are *Morus nigra* (black mulberry) and *Morus mesozygia* (African mulberry). Mulberry fruits are rich in anthocyanins and alkaloids with flavonoids as the major constituent. Mulberry leaves are believed to possess various biological activities, such as antioxidant, antimicrobial, skin-whitening, cytotoxic, antidiabetic, glucosidase inhibition, antihyperlipidemic, antiatherosclerotic, antiobesity, cardioprotective, and cognitive enhancement activities [15, 16]. Our study focused on scientific validation of *Morus mesozygia*, which is claimed to treat DM in western Uganda, by determining its efficacy and toxicity in wistar rats.

## 2. Methods and Materials

### 2.1. Reagents

The analytical grade chemicals used included alloxan monohydrate, distilled water, alpha-naphthol, absolute alcohol, sulphuric acid, copper sulphate, sodium citrate, anhydrous sodium carbonate, cupric acetate, lactic acid, iron (III) chloride, lead subacetate, magnesium ribbon, hydrochloric acid, chloroform, acetic anhydride, ammonium hydroxide, formaldehyde, mercuric chloride, potassium iodide, benzene, glibenclamide, xylene, paraffin wax, hematoxylin, and eosin. These were obtained from Joint Medical Stores, Uganda.

### 2.2. Authentication and Preparation of Plant Extract

The leaves of *Morus mesozygia* were acquired from Ishaka-Bushenyi in western Uganda and authentication was done by Dr. Olet Eunice, a botanist from Mbarara University of Science and Technology who assigned n herbarium voucher number #001(a)*Morus Mesozygia*. The leaves were air-dried at room temperature (24 ± 3°C) for 14 days. After drying, the leaves were ground by mortar and pestle and pulverized using an electric blender. The powdered plant material was extracted in distilled water on a mechanical shaker (Stuart Scientific Orbital Shaker, UK) for 48 hours. The extract was then filtered using a Buchner funnel and filter paper (Whitman No. 1). The resulting filtrate was dried in a hot air oven at 35°C for one week. The harvested extract was weighed and stored in a refrigerator (2–6°C) in amber jars. Before administration, the extract was reconstituted with distilled water and administered according to body weight [17, 18].

### 2.3. Phytochemical Analysis

To test for the presence or absence of classes of various phytochemical constituents, we used methods described by Evans [19].

### 2.4. Experimental Animals

A total of 30 male adult wistar rats of average weight 150 g were purchased from the Pharmacology Department of Kampala International University (KIU). The animals were housed in metabolic cages and left to acclimatize to the environment in the biochemistry laboratory of KIU for two weeks. The temperature was maintained at 22–26°C by opening windows. The rats were fed with commercial rat feed and water ad libitum under environmental conditions of 12-hour light/dark cycle.

### 2.5. Acute Toxicity Studies

The amount of extract required to kill 50% of a given test population, also known as the median lethal dose (LD50) of the leaf extract of *Morus mesozygia*, was determined using the Lorke method [20]. In phase one, nine rats were divided into three groups of three rats each. The first group received the extract orally at a dose of 200 mg/kg, group two received the extract at a dose of 400 mg/kg, while group three received the extract at the dose of 800 mg/kg [21]. The animals were observed for general signs and symptoms of toxicity over a period of 48 hours, which included motor activity, anesthesia, tremors, arching and rolling, chronic convulsions, ptosis, tonic extension, lacrimation, Straub reaction, exophthalmos, piloerection, salivation, muscle spasms, opisthotonus, writhing, hyperesthesia, loss of righting reflex, depression, ataxia, stimulation, sedation, blanching, hypnosis, intestinal distension, diarrhea, lethargy, nasal or anal bleeding, coma, and death. None of the above symptoms were observed, which led to carrying out phase two.

In phase two, 4 rats were divided into 4 groups of one rat each. The extract was administered at the dose of 1,250; 2,500; 5,000, and 10,000 mg/kg orally to groups 1, 2, 3, and 4, respectively, and the animals were observed for general signs and symptoms of toxicity over a period of 48 hours [21]. Based on the result of both phases, the final LD50 was to be calculated as the square root of the geometrical mean of the highest nonlethal dose and the lowest lethal dose [20].

LD_50_ is calculated by using the following formula [20]:

(1) $$\begin{array}{c} {\text{LD}}_{50}=\sqrt{\left(D_{0}\times D_{100}\right)}, \end{array}$$

where *D*_0_ is the highest dose that gave no mortality and *D*_100_ is the lowest dose that produced mortality.

### 2.6. Induction of Hyperglycemia

Hyperglycemia was induced by an intraperitoneal injection of 200 mg/kg body weight of alloxan monohydrate reconstituted in normal saline after an overnight fast of 16 to 18 hours. The rats were then provided with water and food. After three days, blood samples were obtained by the tail snip method and the sugar level of each animal was determined using an “On Call Plus” glucometer. All rats with blood glucose concentration greater than 200 mg/dl were considered hyperglycemic [22].

### 2.7. Experimental Procedure

Study animals were divided into 6 groups of five rats each. Three of the groups received the extract in doses of 200, 400, and 800 mg/kg per body weight; the 4^th^ diabetic group was given 0.5 mg of the standard drug glibenclamide; the 5^th^ group received 2 ml of normal saline (diabetic control), and the 6^th^ group were nondiabetic rats that received 1 ml of normal saline (normal control). The above doses were selected from previous studies as equivalent doses consumed [23, 24].

After feeding overnight, the extract, glibenclamide, and normal saline were administered orally each day for 14 days.

Blood glucose was measured hourly from 0 hour to 4 hours from the time of administration of the extract and control drug. The elimination half-life of glibenclamide is about 4 hours in diabetic induced rats. We measured glucose reduction from 0 to 4^th^ hour in comparison to that with the standard control drug [25, 26].

After the last administration of the extract and control drug (14^th^ day), the experimental animals were fasted for about 12 hours and thereafter anesthetized with diethyl ether, killed, and blood samples collected from cardiac puncture into plain vacutainers for biochemical assays.

### 2.8. Estimation of Liver and Kidney Function Tests

Serum levels of aspartate transferase (AST) and alanine transferase (ALT) were analyzed according to the method described in [27, 28]. Serum levels of urea and creatinine were determined by the Jaffe reaction [29], while total cholesterol and TAGs were determined according to methods described by Carlson and Goldfarb [30].

### 2.9. Animal Necropsy and Tissue Processing

The use of animals in this study was in accordance with the internationally accepted principles for laboratory animal use and care stated in the European Community Guidelines (EEC Directive of 1986; 86/609/EEC).

Each rat was deeply anesthetized with diethyl ether by placing the rat in a tight container with cotton soaked in diethyl ether. After anesthesia, the rat was laid in a supine position on a dissection board with limbs stretched outwards using pins. A sagittal incision was made through the thoracic wall to the abdominal region and the ribs were reflected and held laterally. A 21-gauge needle was inserted into the heart and blood was collected into vacutainer tubes for biochemical analysis. With the rat still in a supine position and limbs pinned outwards, mid-sagittal incisions were made through the anterior abdominal wall into the peritoneum to expose the abdominal viscera. Carefully, the liver and kidneys were removed and immersed in small containers with 10% buffered formaldehyde solution for histological processing.

Histology sections of approximately 0.2 cm × 0.2 cm were cut from the liver and kidneys of each rat. The tissues were dehydrated through descending grades of alcohol to absolute alcohol, then cleared in xylene, infiltrated with paraffin wax, and embedded in molten paraffin wax. Tissue sections were cut at 4 *μ*m with a rotary microtome. Cut sections were mounted on slides, stained with hematoxylin and eosin, and then viewed under a microscope.

### 2.10. Ethical Consideration

Ethical approval and clearance for use of laboratory animals was sought and issued by the Institutional Research and Ethics Committee (IREC) of Kampala International University (MScBCH/0002/72/DU). The guide for the care and uses of laboratory animals was followed and the rats were kept in metabolic cages with adequate light and air. They were fed with food and water as described by the study. On completion of the study, the rats were killed after anesthesia. All animal remains were collected and taken for incineration.

### 2.11. Statistical Analysis

Data were analyzed using GraphPad Prism version 7.03, where mean and standard deviation of means were determined within each group. Two-way ANOVA followed by Tukey's post hoc test was done to test for significance between the experimental groups at 95% confidence interval, where significance was regarded when *P* ≤ 0.05 and nonsignificance when *P* > 0.05.

## 3. Results

The components in Table 1 were identified in a crude leaf extract obtained from *Morus mesozygia*.

### 3.1. Acute Toxicity Study

There were no deaths observed in the acute toxicity tests, and therefore, LD50 could not be calculated. However, writhing was observed in the rats administered with doses of 5000 and 1000 mg/kg, and this led the choice of doses 200, 400, and 800 mg/kg used in the study.

### 3.2. Glycemic Tests

From days 1 to 4, the mean reduction in glucose levels from the 0 to 4^th^ hour was 6.21% in the 200 mg group, 14.72% in the 400 mg group, and 10% in the 800 mg group. The standard control reduction was 35%. There was a significant difference (*P*=0.012) in glucose reduction in the 200 mg group at the 4^th^ hour. No significant difference was observed in the rest of the test groups during the first 4 days as shown in Table 2.

From days 5 to 8, percentage reduction in glucose levels among the test groups was highest among the 800 mg group (37%), followed by the 400 mg group (28%), and lowest in the 200 mg group (15%) as shown in Table 2. The standard drug reduced glucose levels by 24%. There was a statistical difference in the 800 mg group during the 0 and 4^th^ hour (*P*=0.029 and *P*=0.0016, respectively).

From days 9 to 14, glucose reduction was highest in the 800 mg group (65%), 31% in the 400 mg group, and 3% in the 200 mg group. The reduction was 38% with the standard control drug. There was no significant difference in the test groups (*P* > 0.05) as shown in Table 2.

**Figure 1:**
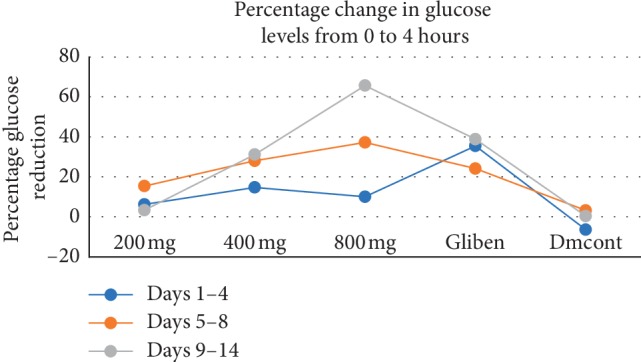
Graph showing percentage changes in blood glucose levels from the study groups. The change was a measure of difference in blood glucose reading from 0 hour reading and 4^th^ hour compared at different days. Gliben = diabetic-induced rats that received the standard drug glibenclamide; Dmcont = diabetic-induced rats that received normal saline.

Percentage glucose reduction in the study groups was lowest in the 200 mg group on days 9 to 14 and highest on days 5 to 8. In the 400 mg group, percentage reduction was highest on days 9 to 14 and lowest on days 1 to 4. In the 800 mg group, glucose reduction was highest on days 9 to 14 and lowest on days 1 to 4 as shown in Table 2 and Figure 1.

### 3.3. Biochemical Parameters

Table 3 shows serum levels of TT protein, albumin, TAGs, cholesterol, AST, ALT, creatinine, and urea from control and treatment groups.

Results shown were recorded as mean ± SD of individual biochemical tests in all experimental animals. Comparisons were made using 2-way ANOVA, showing significant differences.

There was no significant difference in the 200 mg group in all tests (*P* > 0.05), whereas the 400 mg group showed significant differences in the values of total protein when compared with the standard drug (*P*=0.0395). All tests in the 800 mg group showed no significant difference compared with the standard control drug (*P* > 0.05), as shown in Table 4.

**Figure 2:**
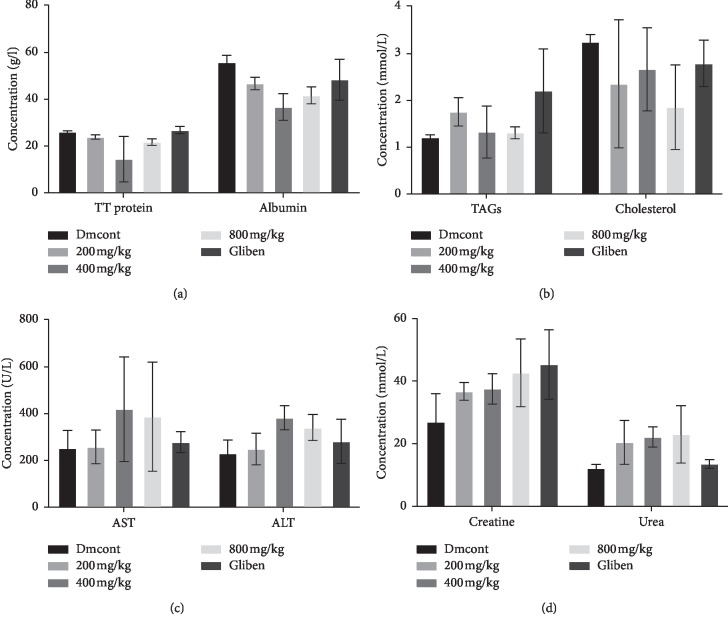
The (a) protein profile, (b) lipid profile, (c) LFT, and (d) RFT, respectively, after treatment of the diabetic induced rats with *M*. *mesozygia* extract. Statistical analysis generally indicated that there was no significant difference in the biochemical profiles of rats treated with the extract and those treated with the standard drug. LFT = liver function test; RFT = renal function test; Gliben = diabetic-induced rats that received glibenclamide; Dmcont = diabetic-induced rats that received normal saline.

Results from Table 3 were compared in different groups in order to ascertain the existence of statistical difference. Data above show *P* values between various experimental groups. Data were significant at *P* < 0.05 (see Figure 2).

### 3.4. Histological Analysis

#### 3.4.1. Liver

**Figure 3:**
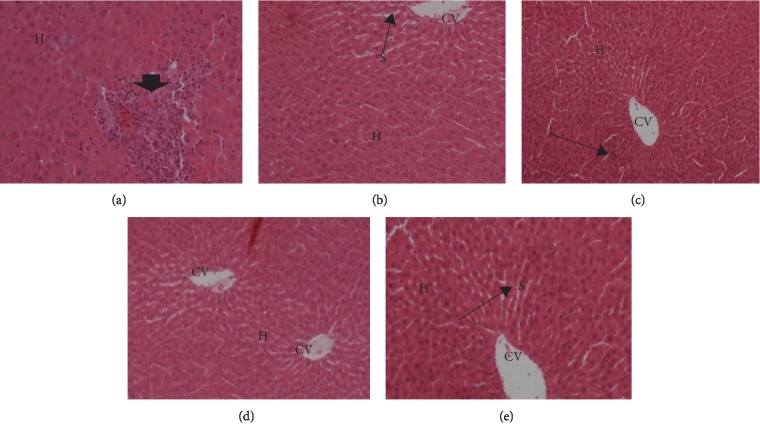
Microphotograph of the liver (H&E, ×200) of alloxan-induced hyperglycemic adult wistar rats exposed to 200 mg, 400 mg, and 800 mg of *Morus mesozygia* for 14 days. In the diabetic control group, the arrow head points at an area of the liver that exhibited focal hepatocellular necrosis with mononuclear cell infiltration (granuloma). These rats died before the 14^th^ day because of the effects of hyperglycemia. There were no observed significant lesions in all the test groups. Note: central vein (CV), hepatocyte plates (H), and sinusoidal spaces (S) are shown in the micrographs stained with hematoxylin and eosin (H&E). (a) Diabetic control group. (b) 200 mg group. (c) 400 mg group. (d) 800 mg group. (e) Glibenclamide control.

Microphotograph of the liver (H&E, ×200) of alloxan-induced hyperglycemic adult wistar rats exposed to 200 mg, 400 mg, and 800 mg of *Morus mesozygia* for 14 days is given in Figure 3.

#### 3.4.2. Kidney

**Figure 4:**
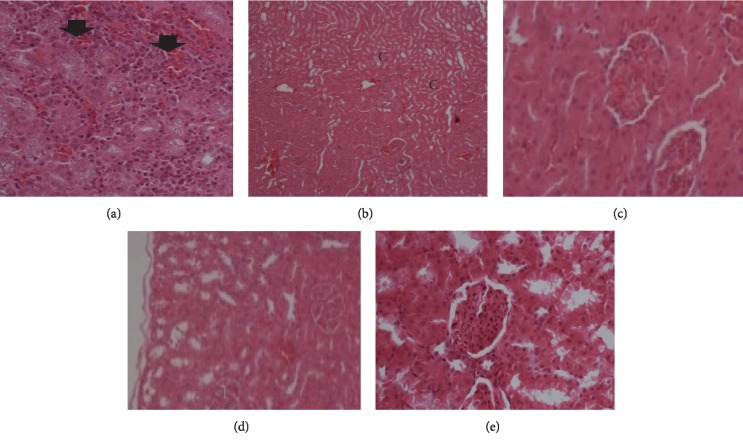
Microphotograph of the kidney (H&E, ×200) of alloxan-induced hyperglycemic adult wistar rats exposed to 200 mg, 400 mg, and 800 mg of *Morus mesozygia* for 14 days. Kidneys of diabetic control rats showed focal necrosis at the corticomedullary junction (black arrow heads). There was mild congestion in the 200 mg group. No significant lesions were observed in the 400 mg and 800 mg groups. Note: C-areas of focal necrosis; H&E = hematoxylin and eosin. (a) Diabetic control group. (b) 200 mg group. (c) 400 mg group. (d) 800 mg group. (e) Glibenclamide group.

Microphotograph of the kidney (H&E, ×200) of alloxan-induced hyperglycemic adult wistar rats exposed to 200 mg, 400 mg, and 800 mg of *Morus mesozygia* for 14 days is given in Figure 4.

## 4. Discussion

This current study aimed at assessing the hypoglycemic and toxic effects of *Morus mesozygia* leaf extract on the liver and kidneys of alloxan-induced hyperglycemic wistar rats.

Under phytochemical analysis (Table 1), reducing sugars were found to be present in large amounts. Reducing sugars increase blood glucose levels in DM due to the absence of insulin that helps in glucose uptake by the cells. This is responsible for the increased blood sugar in the first 4 days of administration of the drug. Proteins were present, with free amino acids in large amounts. Amino acids stimulate the release of insulin from the islets of Langerhans. The release of insulin leads to reduced blood levels of glucose [31]. Therefore, these high amounts of amino acids could have contributed to the hypoglycemic effect of the extract.

Phytochemical analysis also showed the presence of flavonoids, which have been identified as the antidiabetic component of many plants used in herbal medicine [32, 33]. Flavonoids increase glucose breakdown by stimulating increased production of glucokinase mRNA [34]. They also lower the mRNA expression of phosphoenolpyruvate carboxykinase and glucose-6-phosphatase in the liver, thereby lowering the amount of glucose released into circulation [35]. Tannins were found to be present in the extract. Tannins are known to initiate the release of insulin from the pancreas, whose concentration rises in serum and leads to the lowering of blood glucose [33]. Therefore, they are one of the components that contribute to the antidiabetic effect of the extract.

Recent studies have showed that terpenoids lower plasma glucose levels by stimulating the release of insulin and blocking the formation of glucose in the blood stream [36]. Terpenoids were found to be present in moderate amounts in the extract and, therefore, could be a contributor to the hypoglycemic effect of the extract.

On days 1 to 4, the blood sugar of all diabetic groups was above 300 mg/dl, which confirmed their diabetic status (hyperglycemia: blood glucose >200 mg/dl). There was generally a slow decrease in blood sugar from the first hour to the fourth hour (Figure 1 and Table 2), apart from the diabetic control group whose blood glucose level kept increasing due to lack of medication. This high blood glucose was due to the high reducing sugar content of the extract and the low amount of insulin secretion coupled to increased concentration of glucagon that stimulates increased blood glucose.

The rats without any medication (diabetic control) all died within the first three days due to hyperglycemia. This shows the gravity of DM in the absence of medication, which eventually leads to death.

From days 5 to 8, the standard antidiabetic drug glibenclamide reduced blood glucose to slightly below 200 mg/dl, indicating that it had a faster short-term response to reducing blood glucose than the extract, as shown in Figure 1.

The extract administered at 200 mg/kg decreased blood sugar to about 15% at the fourth hour on day 5 but however had a lesser effect towards day 8, indicating that it does not have a longer antihyperglycemic effect.

Administration of the extract at 400 mg/kg gradually reduced blood glucose by 28% at the fourth hour on days 5 to 8; hence, it had a better hypoglycemic effect than the 200 mg dose, indicating that this dose had a long half-life and therefore offered continuous action in a span of 24 hours.

The extract administered at 800 mg/kg was seen to have a little effect (10% reduction) on the reduction of blood glucose in the first 4 days, which is attributed to the high carbohydrate and reducing sugar concentration, but eventually reduced the blood glucose level from days 5 to 8 in the fourth hour by 37% more than the standard diabetic drug at 24% reduction. At this point, the continuous action of the phytochemicals in the extract appears to have stimulated the *β* cells of the islets of Langerhans in the pancreas to release more insulin; increased insulin production leads to lowering of blood glucose.

From days 9 to 14, at 200 mg the extract reduced blood sugar by 3%, while 400 mg and 800 mg doses reduced by 31% and 65%, respectively, as compared to 38% reduction by glibenclamide.

The hypoglycemic effects of the extract at doses 400 and 800 mg/kg could be attributed to the increased insulin secretion activated by the phytochemicals. Another school of thought could be that the extract at doses 400 and 800 mg/kg could have blocked the *α* cell of the islets of Langerhans from producing glucagon hormone, thus preventing stimulation of gluconeogenesis overnight, leading to the hypoglycemia seen at the zero hour before inoculation.

In the biochemical parameter analysis, there was a reduction in albumin levels in the 200, 400, and 800 mg/kg groups, as shown in Figure 2 and Table 3. This could be due to the increased use of proteins in energy metabolism in DM because of the absence of glucose from the cells and breakdown of lipid stores [37]. Diabetes leads to increased levels of plasma glucose, which in turn modify blood plasma proteins by a nonenzymatic reaction called glycation. Protein glycation leads to the formation of toxic molecules called “advanced glycation end products” (AGEs). Accumulation of AGEs has been found to be accelerated in diabetes and contribute to pathogenesis of diabetic complications [38]. Blood plasma proteins are the first to get modified as they are directly exposed to higher glucose concentrations, and a number of them have been identified. Human serum albumin is one of most abundant plasma proteins and heavily glycated in diabetes [39]. Albumin constitutes more than 50% of plasma proteins, and any variation in levels of albumin may change the stoichiometry of glycation of other plasma proteins. Therefore, a drug that is able to maintain low levels of serum and plasma protein is significant.

There was an increase in ALT by the extract at doses of 800 mg/kg, and the 0.5 mg glibenclamide group indicates some damage to the liver, which is in line with [40]. This shows that the extract at a concentration more than 800 mg/kg can be more harmful to the liver.

ALT and AST are both confined in periportal hepatocytes and are known biomarkers of liver damage. Therefore, any increase in serum or plasma levels of these aminotransferases usually indicates damage to the structural integrity of hepatocytes [41]. The extract decreased the level of triglycerides more than the standard diabetic drug; however, the cholesterol level was maintained with no statistical difference compared with the normal control. During metabolism, the pancreas releases insulin which breaks down glucose into energy; however, insulin also allows the body to utilize triglycerides in energy generation [42]. Therefore, a high level of triglycerides indicates that the body cannot convert food into energy. A common cause of high triglycerides is excess carbohydrates in the diet. High triglyceride levels may also designate insulin resistance, which results in higher than normal blood sugar levels [42]. However, our extract was able to reduce triglycerides to levels below that produced by the standard diabetic drug.

Elevated levels of plasma or serum creatinine and urea indicated renal dysfunction in alloxan-induced diabetic rats. Increased plasma urea may indicate reduced reabsorption within the renal epithelium, while high plasma levels of creatinine may perhaps indicate deficiency in the kidneys' glomerular filtration rate [43]. Creatinine and urea are waste products of metabolism; the extract was much better in reduction of these products than the standard diabetic drug, as shown in Table 3 and Figure 2.

From our study, there was no significant change between values of creatinine and urea of the test group and those of the standard diabetic drug. Research has shown that poorly controlled blood sugar levels can cause an increase in the serum urea levels and thus increase the chances of the patient suffering from diabetic nephropathy [44, 45]. Studies have also shown that increased urea and serum creatinine indicate progressive renal damage. It should be noted that urea and plasma creatinine concentration can be imperfect indices of poor glomerular filtration rate because there are other factors that may cause their increase. These include dehydration, gastrointestinal bleeding, high-protein diet, trauma, severe infection, and starvation, among others [44]. Precaution was taken in that healthy animals were chosen in the study and they were adequately fed in order to rule out such causes.

The histopathology slides showed no significant lesions suggestive of any acute toxicity or chronic toxicity in both liver and kidneys, as shown in Figures 3 and 4. The mild congestion seen in some groups was probably due to the use of anesthesia during the killing of the animals.

## 5. Conclusion

From this study, we concluded that *Morus mesozygia* leaf extract has antidiabetic effects at 400 mg/kg to 800 mg/kg doses and that this effect was associated with the phytochemical composition and the extract. The extract was safe at doses below 800 mg/kg on the liver and kidneys of alloxan-induced diabetic wistar rats.

## Figures and Tables

**Table 1:** Phytochemical composition of *Morus mesozygia* leaf extract.

Components	Inference
Alkaloids	Absent
Saponins	Absent
Flavonoids	Present
Reducing sugar	Present in large amounts without heating
Anthraquinones	Absent
Tannins	Condensed tannins present in moderate amounts
Steroids	Absent
Terpenoids	Present in moderate amounts
Protein	Present
Free amino acid	Present in large amounts
Cardiac glycoside	Absent

**Table 2:** Results showing reduction in glucose levels from the 0 to 4^th^ hour during the experiment.

	Extract/control dose	Mean glucose level at 0 hour ± SD (mg/dl)	*P* value (ANOVA)	Mean glucose level at 4th hour ± SD (mg/dl)	*P* value (ANOVA)	Mean glucose reduction from 0 to 4 hours (mg/dl)	Mean glucose reduction from 0 to 4 hours (%)
Days 1–4	200 mg	393.8 ± 27.23	0.991	273 ± 16.23	0.012^*∗*^	23	6.21
	400 mg	312 ± 16.22	0.053	297.2 ± 31.23	0.772	61.75	14.72
	800 mg	381.6 ± 19.34	0.495	386.2 ± 23.45	0.584	46.88	10.05
	Gliben	408.2 ± 42.11	0.006^*∗*^	261.6 ± 11.43	0.84	159.75	35.48
	Dmcont	455 ± 34.22	0.009^*∗*^	472 ± 33.23	0.0084^*∗*^	−12.2	−6.42

Days 5–8	200 mg	371.5 ± 14.23	0.552	231 ± 18.35	0.150	58.25	15.38
	400 mg	266 ± 23.12	0.300	242.2 ± 11.56	0.08	50.65	28.03
	800 mg	386.33 ± 10.12	0.029^*∗*^	416 ± 22.23	0.0016^*∗*^	109.67	37.23
	Gliben	334.67 ± 25.3	0.439	196.67 ± 27.12	0.990	68.17	24.14
	Dmcont	76 ± 6.87	0.753	76 ± 11.5	0.962	2.55	3.19

Days 9–14	200 mg	234 ± 29.23	0.996	236 ± 24.23	0.936	10.53	3.32
	400 mg	73 ± 13.02	0.558	43.4 ± 13.65	0.703	20.11	31.16
	800 mg	163 ± 25.23	0.629	29.3 ± 15.31	0.605	116.44	65.67
	Gliben	285 ± 23.34	0.0042^*∗*^	199.3 ± 35.53	0.298	62.17	38.85
	Dmcont	82.8 ± 14.92	0.114	78.2 ± 12.12	0.015^*∗*^	0.33	0.367

Note that the rats in the Dmcont group died of hyperglycemia. Gliben = diabetic-induced rats that received glibenclamide; Dmcont = diabetic-induced rats that received normal saline; SD = standard deviation. ^*∗*^Statistically significantly different (*P* < 0.05).

**Table 3:** Effect of *M*. *mesozygia* extract on various biochemical parameters tested.

	Nmcont	200 mg/kg	400 mg/kg	800 mg/kg	Gliben
TT protein	26.10 ± 3.56	23.90 ± 0.95	14.47 ± 9.75^#^	21.73 ± 1.42	26.83 ± 1.55
Albumin	55.95 ± 3.65	46.85 ± 2.67	36.74 ± 5.69^*∗*^	41.72 ± 3.64^*∗*^	48.41 ± 8.75
TAGs	1.20 ± 0.78	1.75 ± 0.30	1.32 ± 0.55	1.30 ± 0.12	2.19 ± 0.90
Cholesterol	3.23 ± 0.86	2.34 ± 3.36	2.65 ± 0.88	1.85 ± 0.90	2.78 ± 0.50
AST	252.60 ± 25.33	257.43 ± 23.66	418.07 ± 19.21	386.60 ± 32.22	277.27 ± 18.11
ALT	228.93 ± 7.67	248.27 ± 12.73	381.70 ± 13.67	339.83 ± 19.87	280.73 ± 22.43
Creatinine	27.03 ± 18.92	36.73 ± 2.81	37.57 ± 4.88	42.67 ± 10.80	45.30 ± 11.07
Urea	12.20 ± 2.58	20.46 ± 7.01	22.21 ± 3.25	23.03 ± 9.16	13.60 ± 1.41

^*∗*^Significantly different in comparison with normal control (*P* < 0.05). ^#^Significantly different in comparison with the standard drug (*P* < 0.05).

**Table 4:** Adjusted *P* values showing the relationship between experimental groups.

Tukey's multiple comparisons test	TT protein	Albumin	TAGs	Cholesterol	AST	ALT	Creatinine	Urea
	Adjusted *P* value							
Nmcont vs. 200 mg/kg	0.9803	0.1905	0.8747	0.5427	>0.9999	0.9996	0.4652	0.6140
Nmcont vs. 400 mg/kg	0.0577	0.0009	0.9996	0.8470	0.4432	0.5199	0.3855	0.4347
Nmcont vs. 800 mg/kg	0.8061	0.0145	0.9998	0.1511	0.6376	0.7760	0.0870	0.3584
Nmcont vs. Gliben	0.9997	0.3522	0.4410	0.9303	0.9990	0.9820	0.0345	0.9991
200 mg/kg vs. 400 mg/kg	0.1648	0.1215	0.9433	0.9819	0.4719	0.6411	0.9999	0.9980
200 mg/kg vs. 800 mg/kg	0.9814	0.7004	0.9337	0.9058	0.6677	0.8724	0.8385	0.9911
200 mg/kg vs. Gliben	0.9452	0.9946	0.9337	0.9386	0.9996	0.9969	0.5818	0.7569
400 mg/kg vs. 800 mg/kg	0.3874	0.7237	>0.9999	0.6304	0.9973	0.9919	0.8989	0.9999
400 mg/kg vs. Gliben	0.0395	0.0567	0.5601	0.9994	0.5948	0.8285	0.6690	0.5780
800 mg/kg vs. Gliben	0.7053	0.4669	0.5392	0.4978	0.7847	0.9709	0.9903	0.4923

## Data Availability

The data used to support the findings are all included in the article.
